# Determinants and indicators of successful ageing associated with mortality: a 4-year population-based study

**DOI:** 10.18632/aging.102769

**Published:** 2020-02-06

**Authors:** Wei-Ju Lee, Li-Ning Peng, Ming-Hsien Lin, Ching-Hui Loh, Liang-Kung Chen

**Affiliations:** 1 Aging and Health Research Center, National Yang Ming University, Taipei 11211, Taiwan; 2 Department of Family Medicine, Taipei Veterans General Hospital Yuanshan Branch, Yuanshan Township, Yilan County 264, Taiwan; 3 Center for Geriatrics and Gerontology, Taipei Veterans General Hospital, Taipei 11217, Taiwan; 4 Center of Health and Aging, Hualien Tzu Chi Hospital Buddhist Tzu Chi Medical Foundation, Hualien County 790, Taiwan

**Keywords:** life satisfaction, finance, physical activity, stress, successful aging

## Abstract

Successful aging may be a solution to the major challenges that population aging poses to healthcare systems, financial security, and labor force supply. Hence, we studied the value of factors discovered by exploratory factor analysis in predicting four main indicators of successful aging, and their association with mortality. We followed-up a nationally representative sample of 1284 older adults for a median of 50 months. Successful aging was defined by fast walking, independence, emotional vitality, and self-rated health. Exploratory factor analysis revealed five determinants: physical activity, life satisfaction and financial status, health status, stress, and cognitive function. Physical activity and health status were significant factors in living independently. Life satisfaction and financial status were associated with walking speed. Stress was solely associated with emotional vitality. Life satisfaction and financial status, and health status, were important predictors of self-rated health. Compared to people without any successful aging indicators, those with one, two, three, or four showed dose-dependent lessening of mortality risk, with respective hazard ratios of 0.39 (95% CI 0.25–0.59), 0.29 (95% CI 0.17–0.50), 0.23 (95% CI 0.11–0.51), and 0.09 (95% CI 0.01–0.66). These associations were stronger in males, older adults, smokers, and drinkers, than in their counterparts.

## INTRODUCTION

The outstanding success of 20^th^ century clinical medicine and public health considerably increased longevity and population growth. In contrast, increased longevity combined with lower fertility rates will make the 21^st^ century one of population aging. This demographic transition poses multi-dimensional challenges at individual, society and national levels; specifically, increasing complexity and utilization of healthcare, care burden and costs for individuals and society, and a diminishing labor force, with profound impacts on healthcare and social care systems [1]. Developing and implementing strategies to maximize functional ability in older age and achieve successful aging is a potential solution.

Huge heterogeneity in the health conditions that arise over the time course of chronological age indicates that apparent or biological age may be discordant with actual calendar age [2]. Frailty is a dynamic state that reflects assets and deficits across multiple systems, including physical, psychological and societal dimensions, with adverse health consequences when deficits outweigh assets. As such, frailty may be considered a measure of biological age, in terms of accumulated multi-system deficits [3], and frailty and successful aging to represent opposite sides of the same coin, because they are both defined by functional capacity and share common targets for preventive/health promotion interventions [4].

Developing operational definitions of successful aging is an important but challenging endeavor, due to the multidimensional and complex nature of the aging process. A systemic review identified 105 operational definitions in different studies; although most used biomedical and, increasingly, psychosocial-centered constructs, there is no consensus yet [5]. Despite this lack of consistent measurement tools, key features of successful aging generally encompass physical, psychological and social-engagement dimensions [6, 7]. Pertinently, Mount and co-workers have proposed a pragmatic multi-dimensional concept of successful aging that includes walking speed, dependence, emotional vitality, and subjective health status, and used exploratory factor analysis to dissect associated predictive factors for various successful aging domains [8]. That study established a foundation for further research on successful aging, in terms of either operational definitions or exploring contributory factors across various domains.

Given the quantifiable features of biological aging and functional capacity [2], frailty may be the antithesis of successful aging as operationalized in terms of functional capacity [4]. We previously identified several factors potentially implicated in frailty, including physical activity, life satisfaction, health status, stress, and cognition, and demonstrated an association with mortality [9]. Although these factors may contribute to successful aging, their value in predicting individual dimensions of successful aging is unknown. Hence, we investigated the value of these factors in predicting biomedical and psychological indicators of successful aging, and their impact on mortality, specifically, whether there is a dose-response relationship between numbers of successful aging indicators and mortality.

## RESULTS

This study included 1284 adults > 50 years old from Taiwan who participated in The Social Environment and Biomarkers of Aging Study (SEBAS) [10], representing total follow-up of 5088 person-years. There was a higher likelihood of successful aging among people who were younger, male, had a higher education level, with a lower burden of disease, and who used tobacco and alcohol (Table 1).

**Table 1:** Participant characteristics and demographics.

**Data show mean ± standard deviation or number (%)**	**All**	**Number of successful aging indicators**					
		**None**	**One**	**Two**	**Three**	**Four**	**p value**
Number	1284	107	554	360	193	70	
Age (years)	65.8 ± 9.9	75.7 ± 8.2	67.0 ± 9.7	64.3 ± 9.3	61.7 ± 8.2	59.7 ± 7.3	< 0.001
Male	679 (52.9)	49 (45.8)	279 (50.4)	192 (53.3)	113 (58.5)	46 (65.7)	0.030
Education duration (years)	7.1 ± 4.9	3.8 ± 4.2	6.4 ± 4.6	7.5 ± 4.9	8.8 ± 4.7	10.3 ± 4.6	< 0.001
Smoke	251 (19.5)	7 (6.5)	118 (21.3)	69 (19.2)	40 (20.7)	17 (24.3)	0.008
Drink alcohol	351 (27.3)	14 (13.1)	140 (25.3)	103 (28.6)	67 (34.7)	27 (38.6)	< 0.001
Charlson Comorbidity Index	0.8 ± 1.0	1.5 ± 1.2	0.9 ± 1.1	0.6 ± 0.9	0.4 ± 0.7	0.3 ± 0.6	< 0.001

Table 2 shows the predictive values of five determinants discovered by exploratory factor analysis on four indicators of successful aging, among which dependency had the highest predictive value, as indicated by having the smallest Akaike information criterion and Bayesian information criterion values [11]. Physical activity and health status were significant factors in achieving independent status, besides which, life satisfaction and financial status were also associated with walking speed. Emotional vitality was the only indicator of successful aging significantly associated with stress. Life satisfaction and financial status, and health status, were significant predictors of self-rated health.

**Table 2:** Comparison of factors discovered by exploratory factor analysis with indicators of successful aging.

**Successful aging indicator**	**Fast Walking**		**Independence**		**Emotional Vitality**		**Subjective health**	
**Discovered factor**	**Odds Ratio (95% CI)**	**p value**	**Odds Ratio (95% CI)**	**p value**	**Odds Ratio (95% CI)**	**p value**	**Odds Ratio (95% CI)**	**p value**
Physical activity	0.77 (0.63–0.93)	0.008	0.80 (0.77–0.84)	< 0.001	0.94 (0.89–0.99)	0.011	0.98 (0.95–1.01)	0.126
Life satisfaction and financial status	0.99 (0.99–1.00)	0.013	1.00 (0.99–1.01)	0.617	0.91 (0.89–0.92)	< 0.001	0.99 (0.98–0.99)	< 0.001
Health status	0.99 (0.98–0.99)	< 0.001	0.97 (0.96–0.99)	0.002	0.98 (0.97–0.99)	< 0.001	0.93 (0.92–0.94)	< 0.001
Stress	1.00 (1.00–1.01)	0.723	1.00 (0.98–1.01)	0.505	0.98 (0.97–0.99)	< 0.001	1.00 (0.99–1.01)	0.699
Cognitive function	1.00 (0.99–1.01)	0.796	1.01 (0.99–1.02)	0.338	0.99 (0.98–1.00)	0.153	1.00 (1.00–1.01)	0.284
**Model information**		< 0.001		< 0.001		< 0.001		< 0.001
Akaike information criterion	1061		233		859		1078	
Bayesian information criterion	1103		274		900		1119	
− 2 log likelihood	1046		217		843		1062	

CI, confidence interval.

**Figure 1:**
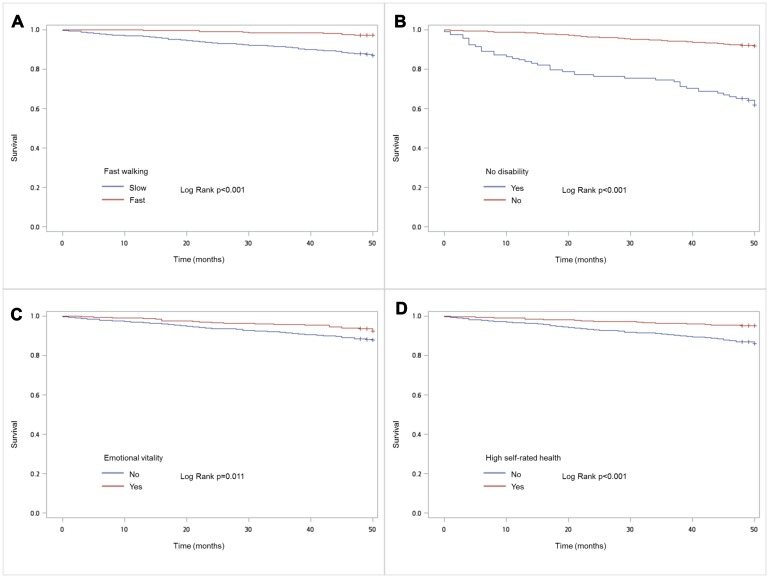
**Kaplan Meir survival plots for domains of successful aging:** (**A**) Fast walking; (**B**) No disability (**C**) Emotional vitality (**D**) Subjective good health.

**Figure 2:**
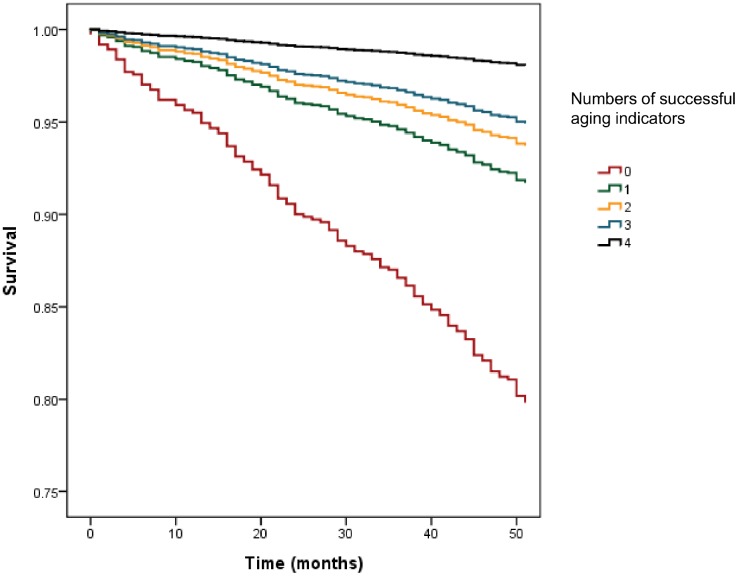
**Adjusted Cox regression analysis for numbers of successful aging indicators on mortality.**

During median follow-up of 50 months, 139 people in the study sample died. Kaplan-Meier analysis showed that the four indicators of successful aging were significantly associated with mortality (Figure 1). Cox proportional hazard models adjusted for age, sex, years of education, smoking, drinking alcohol, and Charlson Comorbidity Index showed a dose-dependent association of increasing numbers of successful aging indicators in lessening the risk of mortality (Figure 2); fast walking speed, no disability, and good subjective health predicted prolonged survival (Table 3).

**Table 3:** Survival analysis for domains of successful aging.

	**Model one^a^**		**Model two^b^**	
	**Hazard ratio (95% CI)**	**p value**	**Hazard ratio (95%CI**	**p value**
**Any successful aging indicator**				
≥ 1 versus 0	0.32 (0.22–0.48)	< 0.001	0.35 (0.23–0.53)	< 0.001
**Number of successful aging indicators**				
None (reference)	1		1	
One	0.37 (0.24–0.56)	< 0.001	0.39 (0.25–0.59)	< 0.001
Two	0.26 (0.16–0.45)	< 0.001	0.29 (0.17–0.50)	< 0.001
Three	0.21 (0.10–0.45)	< 0.001	0.23 (0.11–0.51)	< 0.001
Four	0.08 (0.01–0.58)	0.013	0.09 (0.01–0.66)	0.018
**Individual successful aging indicators**				
Fast walking	0.43 (0.20–0.94)	0.035	0.45 (0.21–1.00)	0.049
No disability	0.34 (0.23–0.50)	< 0.001	0.36 (0.24–0.55)	< 0.001
Emotional vitality	0.74 (0.46–1.17)	0.198	0.80 (0.50–1.28)	0.346
Good subjective health	0.46 (0.28–0.74),	0.001	0.50 (0.31–0.82)	0.006

CI: confidence interval.

^a^Adjusted for age, sex, and years of education.

^b^Adjusted for Model one, plus smoking, drinking alcohol, and Charlson Comorbidity Index.

**Figure 3:**
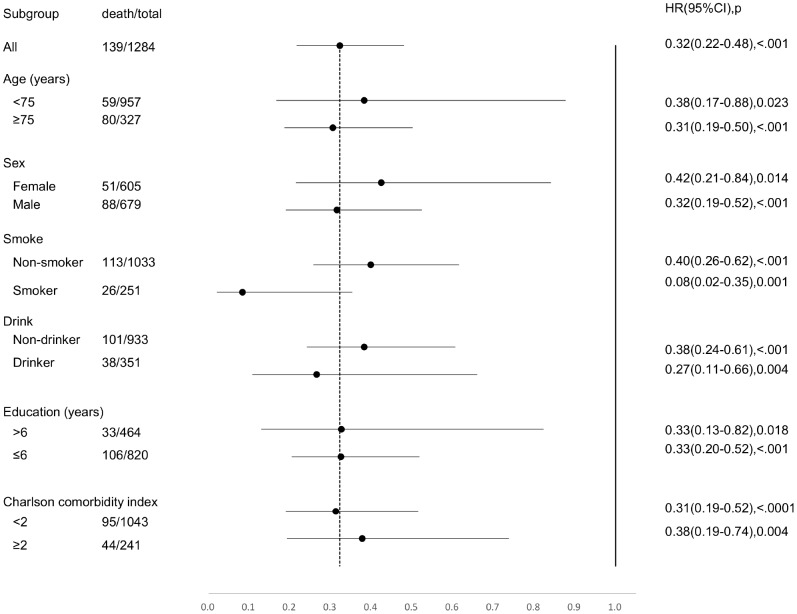
**Forest plot for full adjusted Cox regression analysis of any domains of successful aging against mortality by age, sex, smoking, drinking alcohol, years of education, and Charlson Comorbidity Index.**

Compared to people with none of the four indicators of successful aging, those with any single indicator had 65% lesser risk of mortality (Table 3). These benefits were apparent among males, adults ≥ 75 years old, and people who smoked or drank alcohol (Figure 3).

## DISCUSSION

This nationally representative cohort study discovered a dose-response association between successful aging and protection against mortality. Such associations were stronger in biomedical domains of successful aging than in psychosocial ones. All indicators of successful aging, apart from emotional vitality, were associated with reduced mortality risk.

Our results corroborate evidence of a relationship between successful aging and mortality, consistent with previous studies [12, 13]. In a study of 1370 Finnish nonagenarians, three out of four indicators of successful aging – including physical, psychological and social components – were associated with mortality, with a dose-dependent relationship; at 4-year follow-up, the adjusted hazard ratio for mortality was 0.59 (95% CI 0.41–0.83) among ‘successful agers’ [12], similar to our findings. Our study has gone a step farther in re-affirming the relationship between these domains and mortality beyond ’naturally-selected’ nonagenarians to older adults in general. A study of 3848 Koreans found mortality to be associated with successful aging as defined by seven components that included absence of major diseases, depression, or dependency, high physical and cognitive function, active social engagement, and life satisfaction; non-successful agers had higher mortality risk than that of successful agers (HR 1.69, 95% CI 1.18–2.43) [13]. Despite difficulty in comparing all of these studies directly, due to their differing definitions of successful aging, they nevertheless support the hypothesis that successful aging protects older persons against mortality.

Mount et al. have proposed a model of successful aging that is consistent with both biomedical and psychosocial schools of thought, allows for heterogeneity, and avoids focusing on average tendencies within subgroups by using a factor scoring technique [8]; their study constructed an individual health score to operationalize successful aging and explore the relationships between the discovered factor and various domains of successful aging, and to examine relationships between these domains and mortality.

Walking speed reflects general neuromuscular performance and is not only associated with mortality [14], but is also regarded as a key feature of healthy aging [4, 15]. Fast walking speed implies robust health status, and may be an objective indicator of functional capacity, because neurodegenerative or cardiorespiratory conditions conduce to slower walking speed [4]. Dependency (ie, any deficit in activities of daily living) is another important adverse health outcome for elderly people and is also associated with increased mortality risk [16, 17]. These two biomedical indicators of successful aging had higher value in predicting mortality in our study compared with psychosocial indicators. Among the putative determinants discovered in our previous exploratory factor analysis, physical activity and health status, rather than psychosocial components, were associated with walking speed and dependency.

Emotional vitality is a subjective global indicator of mastery, happiness, and lack of anxiety or depressive symptoms, and was associated with lower likelihood of mortality in the community-based Women’s Health and Aging Study (WHAS) [18]. A study of 6265 American men and women followed-up for an average of 15 years, showed that those with high level of emotional vitality had 19% lesser likelihood of incident coronary heart disease, and significantly lower mortality rates [19]. Although four out of five discovered factors in our study were associated with high emotional vitality, no such association with reduced mortality risk was evident, perhaps due to the relatively shorter follow-up period and better health status of our study sample. Although the WHAS affirmed that emotional vitality had protective survival effects in black and white American women who were disabled [20], it found evidence that emotional vitality was significantly associated with black race [18]. Hence, cultural and urbanization related factors that may substantially influence emotional vitality may explain why we found no statistically significant association with mortality in the SEBAS cohort. Self-rated health is a simple single-item health measure that corresponds to an individual’s global health status and is associated with mortality [21], congruent with our findings.

Comorbidity, age, sex and years of education might reasonably be assumed to play roles in successful aging. However, the association of successful aging with smoking or drinking alcohol was an unexpected finding. This counterintuitive insight might reflect residual confounding or different pathogenesis. A similar paradoxical effect of smoking was observed in patients with acute coronary syndrome [22], and alcohol is known to disproportionately harm people with lower socioeconomic status [23]. Although we used years of education as a surrogate indicator to control for socio-economic status there may have been other confounding factors. If this was attributable to a different pathogenic process, our results could not establish causality.

The complexity and heterogeneity of aging justify a multi-dimensional approach to promoting successful aging [24]. People in our sample with more indicators of successful aging showed a dose-dependent positive relationship with likelihood of survival. Men had higher numbers of successful aging indicators than did women, perhaps reflecting shorter life expectancy and years of disability [25]. Compared to people with none of the indicators of successful aging, the hazard ratio for mortality among 5% with the highest successful aging status was reduced by approximately 90%; people with any one indicator had 65% reduced mortality risk compared to those with none. The hugely important implication for policymakers and health professionals, is that stakeholders must be exhorted to implement strategies to help people attain these four indicators; adding even one indicator would confer a substantial health benefit.

This study had noteworthy limitations. First, mortality is not a good outcome measure for assessing the impact of successful aging in geriatric medicine; nevertheless, it is a widely-used public health indicator, and the study data excerpted from national death registries were high-quality. Second, putative factors were discovered in an exploratory factor analysis of 35-items chosen by a panel of geriatric experts under standard procedures for constructing a frailty index [9]. Although these factors covered major bio-psychosocial domains of successful aging, they did not include anthropometric and biochemical factors that may be closely related to aging. Further research may incorporate such variables into operational models of successful aging.

In conclusion, older persons with indicators of successful aging have a dose-dependent reduction in mortality risk, and this association was stronger among men, older adults, and people who smoked or drank alcohol.

## MATERIALS AND METHODS

### Participants and study design

This population-based cohort study excerpted data from the second wave of SEBAS, a nationally representative aging cohort selected using multi-stage proportional-to-size sampling, which was commenced in year 2000 to explore biological, psychological and social aspects of successful aging. Details of the study design, recruitment and data collection have been published elsewhere [10]. The first and second waves had similar protocols, but the second included additional health behavior and assessments. Briefly, the second wave invited 1659 potential participants between August 2006 and January 2007, and 1284 (77.4%) who responded were interviewed face-to-face at home by trained research nurses.

The study design and procedures conform to the principles of the Declaration of Helsinki. The observational design and reporting format follow STROBE guidelines [26]. All participants provided fully-informed written consent. The Joint Institutional Review Board of Taiwan approved the study protocol.

### Successful aging

Based on proposed operational definitions of successful aging, four major indicators include walking speed, dependency, emotional vitality, and subjective health [8]. Walking speed was defined as the average of two measures from a 3-metre walking test at normal pace [27]; the cutoff defining fast walkers was the highest quintile for walking speed [4]. Katz activities of daily living was used to measure physical function [28]; any limitation of instrumental activities of daily living was defined as dependency. Emotional vitality comprised four components: high level of personal mastery [29], being happy, few depressive symptoms and low anxiety based on the operational definition of Penninx et al. [18]. As there was no complete measure of anxiety, the answer to the question “Do you feel stressed or anxious about own health”, was used to score anxiety, according to previous study [8]. Participants with zero of four negative components were defined as being emotionally vital. Self-rated health was scored using a five-point scale, with very poor, poor, and fair categorized as poor subjective health, and good and very good classified as good [21]. These four indicators of successful aging were summed to represent multi-dimensions of successful aging in a successful aging score that ranged from zero to four, with a higher score denoting more successful aging.

### Mortality ascertainment and follow-up

All participants were followed from the index interview date until 31 December 2010, and data on deaths were acquired from the Taiwan national death registry, held by the Ministry of Health and Welfare.

### Other covariates

Variables associated with successful aging and mortality were chosen based on published studies, and included age, sex, years of education, and smoking tobacco or consuming alcohol during the previous 6 months (both yes or no). Charlson Comorbidity Index was used to indicate the burden of diseases [30].

### Factor scoring

We have already published a frailty index and used exploratory factor analysis to identify essential components of frailty: physical activity, life satisfaction and financial status, health status, stress, and cognitive function (Supplementary Table 1) [9]; as frailty can be considered the antithesis of successful aging based on function-centered medicine [4], and since these factors are consistent with theoretical concepts of successful aging proposed by Bowling et al. [31], we used these factors to investigate associations with indicators of successful aging. We derived factor scoring coefficients from a generated regression model, and multiplied the individual measurements of each participant by the weights of the individual variables to get individual variable scores, all of the resulting scores were then summed to generate an overall score for each latent factor.

### Statistical analysis

All analyses were performed using the SAS statistical package, version 9.4 (SAS Institute, Inc., Cary, NC, USA). A p-value from two-sided tests < 0.05, and 95% confidence intervals not spanning the null hypothesis values were considered statistically significant. Numerical variables were expressed as mean plus/minus standard deviation, and categorical variables were as number (percentage). The Student t test was used to compare numerical differences between women and men, and chi square or Fisher exact tests were used, as appropriate, to compare categorical variables. Crude and multivariable logistic regression were used to analyze the values of factors discovered by exploratory factor analysis in predicting individual indicators of successful aging, including walking speed, independence (no dependency), emotional vitality, and self-rated health.

Cox proportional hazard regression was used to evaluate the association between individual indicators of successful aging, summed numbers of these four indicators and mortality; Schoenfeld residuals were used to test proportionality assumptions in Cox proportional hazard models. Subgroup survival analyses included age (< 75 versus ≥ 75 years), sex (male versus female), education duration (≤ 6 versus > 6 years), smoking (yes versus no), drinking alcohol (yes versus no), and Charlson Comorbidity Index (≥ 2 versus < 2).

## Supplementary Material

Supplementary Table 1
