# Supplementary material for: Determinants and indicators of successful ageing associated with mortality: a 4-year population-based study
Source: Aging (Albany NY). 2020 Feb 6;12(3):2670–9. doi: 10.18632/aging.102769 (PMC7041724; doi:10.18632/aging.102769)
Supplement: Supplementary Table 1 [file aging-12-102769-s001..pdf]

SUPPLEMENTARY TABLE

Supplementary Table 1. Factor scoring coefficients derived from a generated regression model.

Factor one: Physical activity		Factor two: Life satisfaction and financial status		Factor three: Health status		Factor four: Stress		Factor five: Cognitive function	
**Item**	**Coefficient**	**Item**	**Coefficient**	**Item**	**Coefficient**	**Item**	**Coefficient**	**Item**	**Coefficient**
Standing continuously for 15 minutes	0.049	Satisfaction of current living situation	0.215	Multimorbidity	0.294	Stress on one's own finances	0.235	Orientation to time (year)	0.229
Raising both hands over head	0.109	Happy	0.401	Subjective rated health	0.276	Stress on family member's health	0.279	Orientation to time (month)	0.260
Grasping or turning objects with fingers	0.107	Life goes well	0.411	Pain	0.250	Stress on family member's finance	0.341	Orientation to time (date)	0.249
Walking 200–300 meters	0.013	Meeting living expenses	0.144	Health status evaluated by observers	0.262	Stress on family member's job	0.326	Orientation to time (day of the week)	0.196
Climbing 2–3 flights of stairs	0.011	Helpless in dealing with problems of life	−0.089					Orientation (current President)	0.209
Buying personal items	0.058	Subjective socioeconomic status	0.153					Orientation (former President)	0.203
Managing money/ paying bills	0.078								
Riding bus/train by yourself	0.020)								
Doing light tasks at home	0.056								
Bathing	0.115)								
Dressing	0.129								
Eating	0.136								
Getting out of bed/ standing up/sitting in chair	0.129								
Moving around the house	0.126								
Toilet	0.129								
